# REGγ is associated with multiple oncogenic pathways in human cancers

**DOI:** 10.1186/1471-2407-12-75

**Published:** 2012-02-23

**Authors:** Jing He, Long Cui, Yu Zeng, Guangqiang Wang, Ping Zhou, Yuanyuan Yang, Lei Ji, Yanyan Zhao, Jiwu Chen, Zhuo Wang, Tieliu Shi, Pei Zhang, Rui Chen, Xiaotao Li

**Affiliations:** 1 Institute of Biomedical Sciences, East China Normal University, 500 Dongchuan Rd., Shanghai 200241, China; 2 Department of Colorectal Surgery, Xin-hua Hospital, Shanghai Jiao-tong Univerisy, Shanghai, People's Republic of China; 3 Department of Pathology, the Second Chengdu Municipal Hospital, Chengdu, China; 4 Department of Molecular and Cellular Biology, Baylor College of Medicine, One Baylor Plaza, Houston, TX 77030, USA

## Abstract

**Background:**

Recent studies suggest a role of the proteasome activator, REGγ, in cancer progression. Since there are limited numbers of known REGγ targets, it is not known which cancers and pathways are associated with REGγ.

**Methods:**

REGγ protein expressions in four different cancers were investigated by immunohistochemistry (IHC) analysis. Following NCBI Gene Expression Omnibus (GEO) database search, microarray platform validation, differential expressions of REGγ in corresponding cancers were statistically analyzed. Genes highly correlated with REGγ were defined based on Pearson's correlation coefficient. Functional links were estimated by Ingenuity Core analysis. Finally, validation was performed by RT-PCR analysis in established cancer cell lines and IHC in human colon cancer tissues

**Results:**

Here, we demonstrate overexpression of REGγ in four different cancer types by micro-tissue array analysis. Using meta-analysis of publicly available microarray databases and biological studies, we verified elevated REGγ gene expression in the four types of cancers and identified genes significantly correlated with REGγ expression, including genes in p53, Myc pathways, and multiple other cancer-related pathways. The predicted correlations were largely consistent with quantitative RT-PCR analysis.

**Conclusions:**

This study provides us novel insights in REGγ gene expression profiles and its link to multiple cancer-related pathways in cancers. Our results indicate potentially important pathogenic roles of REGγ in multiple cancer types and implicate REGγ as a putative cancer marker.

## Background

REGγ, also known as PA28gamma, 11Sgamma, or PSME3, was first identified as Ki antigen, a nuclear protein targeted by autoantibodies found in sera of patients with systemic lupus erythematosus [1]. It is a member of the 11S family of proteasomal activators that have the ability to stimulate the proteolytic activity of the 20S core proteasome independent of ubiquitination and ATP [1]. Accumulating evidence suggests REGγ is involved in cancer progression [2]. REGγ has been reported to be overexpressed in colorectal cancer [3] and thyroid cancer [2], and is involved in cancer development [2,4-6]. It is unknown, however, whether REGγ is involved in additional cancers. REGγ is known to degrade both oncogenic and tumor suppressing proteins such as SRC-3, HCV core protein, PTTG1, p21, p16, p19, and p53. In this study we try to understand expression profiles of REGγ in multiple cancer types and correlations of REGγ with known cancer or cancer related pathways.

Microarray assays have been widely adopted in cancer marker exploration and expression profiling of tumor genes [3,4]. Microarray studies have contributed valuable information to our understanding of cancer by identifying biomarkers and enabling classification of tumor subtypes [5-8].

In this study, we focused on thyroid cancer, colon cancer, liver cancer and lung cancer since the first two cancers were reported with over-expression of REGγ [3,9] and the other two are among the list of the most malicious cancers. We analyzed REGγ expression in cancer tissue arrays by using publicly available microarray data from NCBI GEO database. We acquired datasets and integrated the analyzed results across different datasets and cancer types to characterize a general REGγ expression pattern in four different cancer types by comparing human cancer versus normal tissues. We set clear criteria along with quality controls for dataset screening and normalization, which allowed us to carry out comprehensive dataset-based meta-analysis across differing cancers. A set of genes highly correlated with REGγ expression were identified and validated by RT-PCR to identify putative functional interactions associated with REGγ.

## Methods

### Cell types and cell culture

A549, HepG2, and HCT116 cells were purchased from ATCC and maintained at Cell Culture Core at the Department of Cell Biology, BCM. The human thyroid carcinoma cell line ARO was kindly provided by Dr. Adel El-Naggar at the University of Texas M.D. Anderson Cancer Center. The ARO cell line was authenticated at Genotyping Center of John's Hopkins University. The shN and shR stable cell lines were generated in ARO, A549, and HCT116 by introducing retroviral shRNA vectors specific for REGγ or a control vector from OriGene (Rockville, MD). ARO cells were cultured in 1640 supplemented with 10% fetal bovine growth serum (GIBCO). All other cells were cultured under standard conditions described by the ATCC.

### Immunohistochemical assay

IHC analysis was performed to analyze REGγ expression of protein level in several human cancers including lung, colon, thyroid and liver cancer. Sections were deparaffinised and rehydrated. The slides were then heated in a 100°C water bath for 30 minutes in a 0.01 M citrate buffer solution at pH 6.0, and cooled to room temperature. After quenching the endogenous peroxidase activity with 0.3% H2O2 (in absolute methanol) for 10 minutes, the sections were treated for 10 minutes at room temperature with the serum albumin (HISTOSTAIN-PLUS DAB kit) to block non-specific staining. Duplicate sections were incubated overnight in 4°C with the primary specific antibodies. Slides were then incubated for 10 minutes with biotinylated anti-rab-bit IgG (DAB kit) for REGγ recognition. The sections were incubated with the HRP for 10 minutes. Finally, the sections were counterstained with Mayer's haematoxylin.

### Preliminary datasets collection

Microarray expression profiles were obtained from Gene Expression Ominibus of National Center of Biotechnology Institute [10]. All datasets in this study were published within the past 5 years (2004-2009) and following the Minimum Information about a Microarray Experiment (MIAME) guidelines, including 49 datasets, with 3,832 samples containing 16, 15, 11, and 7 datasets from colon, liver, lung and thyroid cancer, respectively (Additional file 1: Table S2). The following preliminary datasets were retrieved: a) primary tumors, carcinoma and adenoma along with normal controls in each tissue: primary colon cancer samples including early onset colorectal carcinoma, colon tumor and adenoma; primary hepatocelluar carcinoma (HCC); lung cancer including non-small cell lung cancer, adenocarcinoma, and squamous cell carcinoma; thyroid cancer samples including papillary thyroid carcinomas (PTC), anaplastic thyroid carcinoma (ATC), follicular carcinomas (FC) and follicular adenomas. b) non-cancer diseases originated from colon, liver, lung and thyroid tissues, including inflammatory bowel disease (IBD), Crohn's disease (CD), ulcerative colitis (UC), HCV cirrhosis, HCV-induced dysplasia, pneumonia, and follicular goiter. c) different stages of cancers with a stage 0 tissue or healthy or distant adjacent tissues as control. The following datasets/samples were excluded: 1) datasets with no contorl tissue; 2) datasets without REGγ probe/probe set included in platform; 3) datasets without corresponding publication; 4) datasets with samples collected in time courses; 5) datasets without gene symbol annotation for the probes by the Human Gene Nomenclature (HGNC) guidlines; 6) datasets without REGγ data in the microarray platform. The preliminary sample screening yielded 23 datasets (n = 1,070) for differentially expressed gene analysis (Additional file 2: Table S3). Of these output, there were 15 cancer datasets (n = 614), 2 non-cancer diseases datasets of (n = 86), and 6 datasets (n = 399) containing both cancer (n = 260) and disease samples (n = 139).

### Dataset based expression analysis

Microarray datasets (described above) were analyzed by GEOquery and Limma packages in R http://www.r-project.org/ as described previously [11-13]. First, all raw data were downloaded from GEO and mono-channel data were normalized using MAS5.0. Samples in each dataset were grouped into three classes, namely cancer, non-cancer disease and normal samples. The log2 ratio values of disease group versus normal group were calculated based on the normalized data. For all two-channel datasets, log2 transformed expression ratios were calculated and used in all subsequent analyses. Two-sample paired t-test [14] were carried out between cancer vs. non-cancer diseases and cancer vs. normal following statistic analysis as described [15,16]. Internal quality controls were set up for each dataset by validating the statistical significance of specific genes with what was reported in relevant publications. Two-sample comparisons were statistically analyzed for all 21 cancer datasets containing 874 cancer samples and 625 paired normal samples. An additional two-sample comparison was performed with the 8 non-cancer disease datasets, including 196 non-cancer disease samples and 174 normal control samples (Additional file 2: Table S3).

### Correlation analysis

The Pearson's Correlation Coefficient (PCC) was used as a measure of correlation between REGγ and its potentially related genes based on 13 datasets, 4 from liver (n = 299) and 3 from each of lung (n = 164), colon (n = 77), and thyroid (n = 126) respectively. Pearson Correlation analysis was conducted using R [15,17] on datasets with significant overexpression of REGγ. PCC of REGγ with each gene in each dataset was calculated. Genes whose expression correlated with REGγ in each dataset were ranked based on their p-value. In order to produce at least 600 candidates in each datasets for subsequently selection, we used a cutoff of 0.001. The top 20%, 15%, 10%, and 5% genes were selected from thyroid, colon, liver, and, lung cancer datasets respectively. All subsequent selections and analyses were based on these genes referred to as REGγ correlated genes.

Genes were selected from all REGγ correlated genes based on cancer type except for the initial pilot testing (a PCC cutoff as ± 0.6 [18]). Our criteria were that each gene was present in at least 2 datasets, according to binomial distribution (*p *< 0.05), in one cancer type and the cutoff of PCC in one cancer type was set to ± 0.6. Genes that fulfill these criteria were considered as highly-correlated with REGγ and used for downstream pathway analysis.

### Pathway and network analysis

Genes highly correlated with REGγ were analyzed with the IPA (Ingenuity Pathway Analysis) system: http://www.ingenuity.com. With core analysis, all qRT-PCR validated REGγ-correlated genes were mapped and then analyzed using Ingenuity Knowledge Base (genes only) to yield bio-function pathway annotation and networks showing direct and indirect relationships between genes and molecules.

To calculate the composition of REGγ-correlated genes/pathways in cancers, results from Ingenuity pathway analysis were grouped into three clusters: cancer pathways, cancer related pathways, and other pathways. These pathway clusters were grouped based on the following characterization: 1) cancer pathways included bio-function of cancer, tumor or tumorigenesis, neoplasia, carcinoma or adenocarcinoma, lymphoma and sarcoma; 2) cancer related pathways included a) pathways related to cell cycle with following bio-function category: mitosis or mitotic, G2/M/S phase, cell division, check point, and arresting; b) cell growth pathways involved in: survival, growth and proliferation; c) cell death pathways with bio-function of apoptosis and death. 3) Other pathways: all the rest of the pathways not included in cancer or cancer-related pathway clusters.

Genes highly correlated with REGγ were also searched against the KEGG pathways database http://www.genome.jp/kegg/tool/colorpathway.html to highlight and augment the published graphical pathways analyzed by Ingenuity. Protein-protein interaction network analysis was performed by checking REGγ highly-correlated genes in the STRING database http://string-db.org) [19]. To make the network concise, genes with connections equal or greater than 3 were selected.

### PCR validation

Confirmatory qRT-PCR was performed on randomly selected REGγ correlated genes. Fifteen genes were selected from the REGγ-correlated genes and an additional fifteen genes highly correlated with REGγ expression were selected for qRT-PCR. RT-PCR experiments were carried out in cells originated from colon, liver, lung and thyroid cancer.

### RNA preparation, qRT-PCR and RNAi

Cells were grown to 75% confluence in a 6 cm dish and lysed with buffer provided in RNA extraction kit (TAKARA) and RNA was extracted following the manufacturer's instruction. RNA quality and integrity were verified by gel electrophoresis. Two-microgram of total RNA was reverse transcribed with M-MLV reverse transcriptase (Invitrogen). Gene-specific primers were designed as follow: REGγ forward primer, 5'-TCCTCACCAATAGCCACG-3'; REGγ reverse primer, 5'-CTCGATCAGCAGCCGAAT-3'; 18S rRNA forward primer, GGACACGGACAGGATTGACA; 18S rRNA reverse primer, GACATCTAAGGGCATCACAG. qRT-PCR was conducted using the SYBR^® ^PrimeScriptTM RT-PCR Kit (TAKARA). The results were analyzed using the MxPro qRT-PCR software.

RNA interference was performed in HepG2 cells to knock down REG. HepG2 was seeded in 6-well plate at 60% confluence overnight and was transfected with 10 nM siRNA along with lipofection 2000. Cells were harvested 72 hours later for RNA extraction and qRT-PCR analysis.

### Statistic analysis

Weighted student t-test for two-sample with unequal variance was used to calculate statistics and p values for IHC tissue array [16]. Two-tailed student's t-test was used in microarray expression analysis and Fisher's Z transformation was used to adjust p value. A p-value of less than 0.05 was defined as "significant" for all statistic analysis involved in expression analysis. Datasets in which REGγ is highly significant (*p *< 0.001) [18] were selected for subsequent correlation analysis. In correlation analysis, Pearson's correlation coefficient was set with a cutoff PCC ± 0.6 [18] and binomial coefficient was used based on datasets number in each cancer type to selection REGγ highly related genes. Pathways with a p-value less than 0.01 were chosen to be studied in Ingenuity core analysis.

## Results

### REGγ protein is highly expressed in multiple cancers

**Figure 1:**
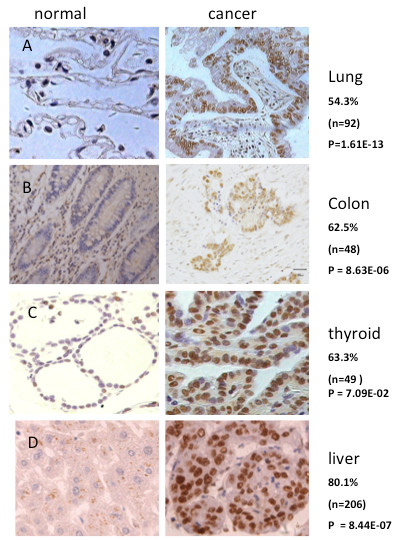
**REGγ protein is highly expressed in multiple human cancers**. A representative result of REGγ overexpression in Human lung (A), colon (B), thyroid (C), and liver (D) carcinoma were demonstrated following IHC experiments. Note that REGγ is only modestly expressed in corresponding normal tissues.

To understand whether REGγ is a tumor-associated protein, we examined REGγ expression levels in multiple human carcinomas. IHC experiment was performed using tissue arrays containing 92 cases of primary lung cancer, 48 colon cancers, 49 thyroid cancers, and 206 liver cancer samples along with corresponding normal tissues, all arranged in duplicates (Table 1, Additional file 3: Table S1). The expression of REGγ in cancer samples was scored double-blindly by comparing with normal tissues or adjacent non-cancer tissues which have no positive staining or low levels of REGγ staining (Figure 1 and Table 1). The scored REGγ expression is consistent for most of the duplicate samples and a representative scored result was shown in Additional file 3: Table S1. The overall rate of REGγ overexpression (a combination of ++ and +++ staining) in different carcinoma is higher than 50%. We observed a statistically significant increase in the number of late-stage cancers with the highest REGγ expression (+++), such as in stage III of adenocarcinoma and squamous cell carcinoma (Additional file 3: Table S1). Our results provide the first evidence for an association of REGγ with primary human lung carcinoma and liver cancer, substantiating previous observations that REGγ is increased in colon and thyroid cancers.

**Table 1:** Summary of IHC analysis of REGγ expression in multiple human cancer tissue

			**REGγ Level**			**p value**
**Cancer type**	**Sample** **Amount**					
		
		-	+	++/+++	-v.s +	-v.s ++/+++
**lung cancer**	**92**	**8 (8.7%)**	**34 (37.0%)**	**50 (54.3%)**	**3.70E-04**	**i.61E-13**
						
**colon cancer**	**48**	**2 (4.2%)**	**16 (33.3%)**	**30 (62.5%)**	**1.74E-04**	**8.63E-06**
						
**thyroid cancer**	**49**	**2 (4.1%)**	**16 (32.7%)**	**31(63.3%)**	**1.44E-01**	**7.09E-02**
						
**liver cancer**	**206**	**5 (2.4%)**	**36 (17.5%)**	**165 (80.1%)**	**5.68E-03**	**8.44E-07**
total	395	17 (4.3%)	102 (25.8%)	276 (69.9%)		

REGγ expression status was scored according to description in Method & Materials. Overexpression rate of REGγ in each cancer was calculated based on the number of cases scored ++ and above. - v.s + represents two sample weighted student t-test for unequal variance between - group and + group. For - v.s ++/+++, it's between - group and ++/+++ group

### Integrated analysis of microarray datasets revealed overexpression of REGγ in selective cancers

**Figure 2:**
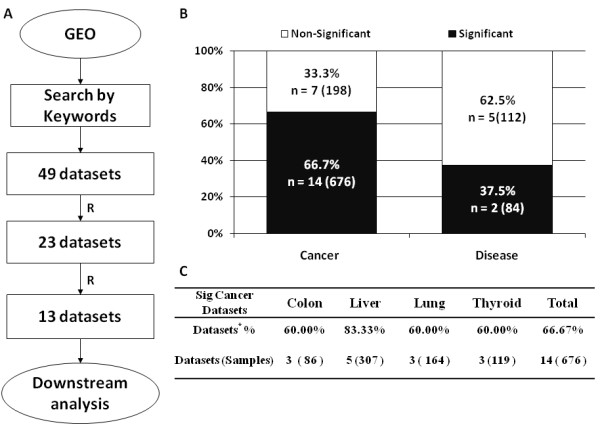
**Microarray meta-analysis of REGγ expression in human cancers**. (A) The flow chart of datasets selection. (B) REGγ expression profiles in Cancer (vs. non-cancer disease datasets. The black boxes refer to the percentage of datasets (14 out of 21 in cancer vs. 3 of 8 in non-cancer datasets) with significant change (*p *< 0.05) of REGγ expression. White boxes represent the percentage of datasets with insignificant changes (7 of 21 in cancer vs. 5 of 8 in non-cancer). (C) REGγ expression profiles in each cancer types.

Overexpression of REGγ protein in four different human cancers prompted us to investigate whether elevation of REGγ is regulated at the mRNA level. We searched GEO database by keywords and identified 49 datasets (Additional file 1: Table S2), of which 23 were qualified for expression analysis in this study (Figure 2A). Significantly higher REGγ expression (p < 0.05) was observed in 67% of cancer datasets (14 of 21, n = 597) when compared with normal tissues (Figure 2B). Consistently, our comparative analysis of control vs. non-cancer diseases [20-22], revealed that most of the non-cancer datasets had no significant differences in REGγ expression (66%, n = 117). On the contrary, only small percent of cancer datasets (7 of 21, n = 228) had no significant elevation in REGγ levels (Figure 2B), indicating potential association of REGγ in the development of these cancers. Cancer type based analysis indicated an increase of REGγ in 60-83% of cancer datasets (Figure 2C), concordant with our IHC studies (Figure 1).

**Figure 3:**
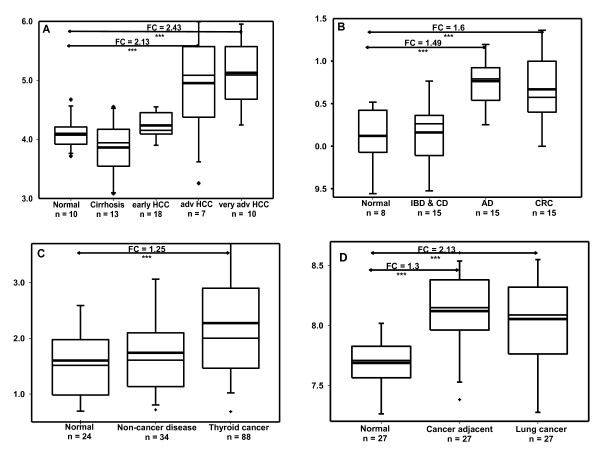
**REGγ expression values and variability in classified human cancers**. Representative REGγ expression fold-change values in pathologically classified, stage-specific cancers, non-cancer diseases and normal control datasets originated from liver (A), colon (B), thyroid (C) and lung (D) tissues were shown in box plot which signifies the upper and lower quartiles. The median is represented by a thin line and mean is represented by a bold line. The upper and lower limit refers to 95% and 5% data values. *** refers to p *<*0.05, * denotes p *>*0.05. FC: fold change between two groups, HCC: hepatocelluar carcinoma, IBD: inflammatory bowel disease, CD: Crohn's disease, AD: Adenocarcinoma, CRC: Colorectal carcinoma.

A detailed analyses of pathologically classified, stage-specific cancers and non-cancer diseases were executed using dataset GSE6764 [23], GSE4183 [22], GSE6339 [21] and GSE7670 [20], which originate from liver, colon, thyroid, and lung respectively and disclosed detailed cancer stage information (Additional file 2: Table S3). The representative REGγ expression patterns in the four cancers, non-cancer diseases, normal controls, and some staged cancer samples are illustrated in Figure 3. Statistically significant elevation of REGγ gene expression in cancers ranged from 1.25 to 2.43 fold-change (overexpression cut-off: fold change > 1), consistent with our IHC result in corresponding cancer tissue arrays (Figure 1). In liver cancer samples, REGγ appeared considerable up-regulation, consistent with the original publication where potential cancer biomarkers were linked with stepwise carcinogenic process [23]. The results of stage-associated increases of REGγ in advanced liver cancers in Figure 3A is consistent with our observation of higher REGγ staining in advanced cancer types. For most of the non-cancer datasets, the p-values of disease classes, such as IBD & CD, showed no significant changes in REGγ expression. In conclusion, expression of REGγ is significantly increased in multiple human cancer types and likely involved in late-stage cancers.

### Correlation analysis links REGγ to large numbers of cancer related genes/pathways

To explore potential mechanisms of REGγ in cancer development, we further investigated genes whose expression is highly related to REGγ expression in the four cancer types profiled. A statistical meta-analysis based on Pearson correlation coefficient (PCC) was conducted on the defined (REGγ differential expressed) datasets (Additional file 4: Table S4). The correlation between REGγ and every other gene in these datasets were calculated and evaluated statistically. With the assumption that a high absolute PCC value would reflect a potentially close relation to REGγ functionally, only genes bearing high PCC scores were selected for subsequent studies.

To estimate that the approach we used in our analysis could indeed generate meaningful results, we first set up a PCC cutoff value +/- 0.6 in at least one dataset for a pilot test. Since previous study [24,25] has demonstrated REGγ mediated regulation of p53, we examined if p53 targets can be identified among REGγ highly correlated genes. A total of 29 published genes in p53 signaling pathway were identified as significantly correlated with REGγ (Additional file 5: Table S5), indicating that our normalized datasets contain valuable information required for further analysis.

**Figure 4:**
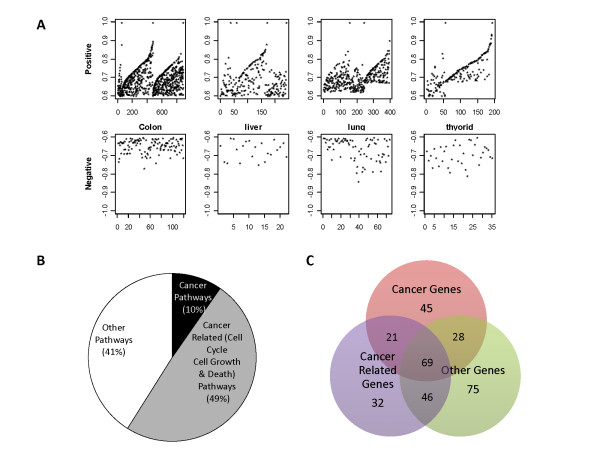
**Statistical analysis and functional annotation of genes correlated to REGγ**. (A) Distribution of PCC (Pearson correlation coefficient) in datasets from different cancers. Upper panel box-plot shows positive PCC and lower panel displays negative PCC values (Y axis) that are greater than +0.6 or less than -0.6. Analyzed datasets are from colon (n = 3), liver (n = 4), lung (n = 3) and thyroid (n = 3). (B) Most of the annotated REGγ-correlated gene pathways are involved in cancers. Ingenuity analysis of REGγ-correlated gene pathways were grouped into cancer (black, 10%), cancer related (grey, 49%) and other pathway clusters (white, 41%) to reveal the proportion of REGγ-correlated gene pathways in cancers. (C) Most of the annotated REGγ-correlated genes are cancer-related. The genes representing cancer, cancer related and other-pathway clusters were plotted to show the overlaps among different pathway clusters. Note that the total number of cancer and cancer-related genes constitute majority of the REGγ-correlated genes.

By more stringent PCC-value based criteria, we screened genes highly-correlated with REGγ and identified a total of 588 genes, with 521 positively correlated, 99 negatively correlated, and 31 being both negatively and positively correlated (Additional file 6: Table S6). Among these genes strongly correlated with REGγ, 467 were identified from colon cancer, while 75, 53, and 25 genes were from lung, thyroid and liver cancer respectively. Interestingly, multiple cancers shared significant amount of these genes. Based on all calculated results, there were 32% genes in two cancers, 43% genes in three cancers and 21% genes in four cancers simultaneously (Additional file 7: Figure S1A). The PCC between REGγ and all other genes in each dataset are shown in Figure 4A. The range of the PCC plot reflects more positive-correlation points than negative ones, suggesting that more genes are positively correlated with REGγ expression.

To understand functional diversities of the genes computationally correlated with REGγ, we performed Ingenuity pathway analysis of the 588 genes. Our analysis displayed that all mapped genes were functionally annotated into 500 pathways in which 207 were statistically significant (*P <*0.01). Among the 207 pathways analyzed, 20 cancer pathways (10%), 102 cancer related pathways (49%), and 85 other pathways (41%) were classified (Figure 4B). The top 15 pathways based on statistic significance (p-values) are shown in Additional file 7: S1B. Cancer related pathways were composed of 86 pathways related to cell cycle regulation, 9 pathways in apoptosis, and 7 pathways in cell growth (Additional file 6: Table S6). Due to the presence of subset of genes in multiple pathways, such as Myc (in 37 function annotation pathways), HSP90AB1 (in 11 pathways), ILF2 and ODC1 (in 6 pathways respectively), the number of genes in cancer, cancer-related and other pathways were 163, 168, and 218 respectively with overlaps indicated in Figure 4C. Detailed information of pathway analysis is included in Additional file 6: Table S6. Based on Ingenuity analysis of cancers with REGγ overexpression, our results indicate that over 50% of REGγ highly-correlated genes/pathways are cancer or cancer-related.

We also validated our pathway analysis of REGγ-correlated genes by applying all 588 REGγ highly correlated genes to KEGG pathway annotation. The results were consistent with Ingenuity analysis whereby cell cycle and cancer pathways were ranked among the top (Additional file 8: Table S7). Based on these annotation analyses, we discovered that REGγ is linked to large numbers of cancer related genes, including Myc & RAN in oncogenic pathway, BUB3 in spindle check-point function, BTG2 in cell cycle transition, DDB1 in DNA damage repair, DAPK2 in programmed cell death, in addition to genes in the p53 pathway like PTEN. We also observed that proteasome, ubiquitin-mediated proteolysis, and metabolic pathways were listed among the top of the 110 pathways covering 125 genes. Gene signaling pathways identified in KEGG analysis also include MAPK, Wnt, Jak-STAT, Neurotrophin, TGF-β, mTOR, and VEGF pathways. A battery of interesting genes were observed in the "other pathways" cluster, encompassing genes in spliceosome like HNRNPC & SFRS3, genes in aminoacyl-tRNA biosynthesis such as DARS & KARS, genes in immune response containing TNFSF10 & MET, as well as genes involved in epigenetic regulation, including SUV39H1, H2, PRMT5, etc.

To illustrate potential links between the gene products among the REGγ correlated genes, we conducted further analysis of protein-protein interaction (PPI) network using STRING [19], which is an online database of known and predicted protein interactions. This generated network (Additional file 9: Figure S2) integrated information from experimental repositories, computational prediction and published collections, and showed their interaction with default parameters. PPI network revealed potential interactions among five clusters of REGγ correlated gene products, including those in metabolic pathways, proteasome pathways, cell cycle related pathways, DNA repair pathways, and tRNA biosynthesis pathways. These results provide additional information for future study of cellular function of REGγ as well as its regulation.

### Confirmatory analysis of REGγ correlated genes from bioinformatic analysis

Our computational analysis indicated strong correlation of REGγ to genes regulated by p53 and in cancer related pathways. To validate our bioinformatics-based predictions, we selected 30 genes for expression analysis using specific cancer cell lines. In addition to genes associated with p53 pathways, we selected two representative genes from each of the top cancer/cancer related pathways, metabolic pathways as well as those appeared in KEGG and Ingenuity network analysis (Table 2). We made use of stable cell lines constitutively expressing a control shRNA (shN) or a REGγ specific shRNA (shR). Three pairs of shRNA expressing cell lines were originated from lung, colon, and thyroid (A549, HCT116, and ARO, Additional file 10: Figure S3). The REGγ knockdown in HepG2 liver cancer cell lines was generated by introducing synthetic siRNA against REGγ [26]. The significant differences of REGγ expression between control (shN) and REGγ knockdown in each pair of the cell lines allowed us to examine the relative levels of genes predicted to be highly correlated to REGγ (Additional file 10: Figure S3). Real time RT-PCR was performed with specific primers for each gene in multiple pairs of cell lines or in a specific pair of cells dependent upon cancer-type specific bioinformatics data (Additional file 11: Table S8).

**Table 2:** A summary of confirmatory qRT-PCR analysis of selective genes

Tissue	Gene Symbol	PCR value	p-value	Status	Gene Annotation
Colon	BTG2	1.25	1.5E-02	Consistent	A member of the BTG/Tob family
Lung	DAPK2	1.35	6.0E-12	Consistent	Death-associated protein kinase 1 (DAPK1)
Lung	GADD45B	1.63	2.6E-02	Consistent	Growth arrest and DNA-damage-inducible
Lung	SATB1	2.82	4.3E-04	Consistent	SATB homeobox 1
Thyroid	ABCA1	1.68	2.8E-02	Consistent	ATP-binding cassette, sub-family A
Thyroid	B3GALT4	1.32	2.0E-03	Consistent	UDP-Gal:betaGlcNAc beta 1,3- galactosyltransferase
Thyroid	PTEN	1.29	4.3E-02	Consistent	Phosphatase and tensin homolog, tumor suppressor
Colon	CCT3	0.62	2.9E-03	Consistent	Member of the chaperonin
Colon	DKC1	0.60	8.3E-04	Consistent	Dyskeratosis congenita 1, dyskerin
Colon	HSP90AB1	0.82	2.4E-02	Consistent	A member of heat shock
Colon	MYC	0.57	6.7E-03	Consistent	myelocytomatosis viral oncogene homolog
Colon	ODC1	0.80	1.3E-02	Consistent	A p53 target negatively regulated.
Colon	RRM2	0.66	4.0E-02	Consistent	ribonucleotide reductase M2
Liver	DDB1	0.63	3.6E-02	Consistent	Damage-specific DNA binding protein 1
Liver	HN1	0.65	1.3E-03	Consistent	Hematological and neurological expressed1
Liver	ILF2	0.79	5.7E-04	Consistent	Interleukin enhancer binding factor 2
Liver	RAN	0.75	1.3E-02	Consistent	ras-related nuclear protein
Lung	BUB3	0.82	8.2E-03	Consistent	Budding uninhibited by benzimidazoles 3 homolog
Lung	USP14	0.74	2.7E-03	Consistent	Ubiquitin specific peptidase 14
Thyroid	ATR	0.47	5.6E-03	Consistent	Ataxia telangiectasia and Rad3 related
			Total	Consistent	N = 20 (66.7%)
Conlon	ACLY	1.40	3.7E-05	Inconsistent	ATP citrate lyase
Thyroid	TSC2	2.18	1.4E-05	Inconsistent	Tuberous sclerosis 2, tumor suppressor
Colon	CDK1	0.50	7.3E-07	Inconsistent	Cyclin-dependent kinase 1
Colon	SPPL2A	0.38	3.5E-02	Inconsistent	Signal peptide peptidase-like 2A
Conlon	TUBG1	0.43	8.5E-05	Inconsistent	Tubulin, gamma 1
Liver	EHHADH	0.61	4.4E-02	Inconsistent	Enoyl-CoA, hydratase/dehydrogenase
Liver	CYP4F2	0.70	3.2E-02	Inconsistent	Cytochrome P450, family 4, subfamily F
Lung	STARD8	0.66	7.5E-03	Inconsistent	A subfamily of Rho GTPase
Lung	UNC13A	4.73	8.2E-06	Inconsistent	Unc-13 homolog A
Thyroid	PPAP2A	0.63	2.8E-02	Inconsistent	Phosphatidic acid phosphatase type 2A
			Total	Inconsistent	n = 10 (33.3%)

PCR value was the expression level of qRT-PCR results in shR (REGγ depletion) cells relative to that in shN (REGγ expressing) cells (see Figure S4). The results were averaged from three independent experiments

**Figure 5:**
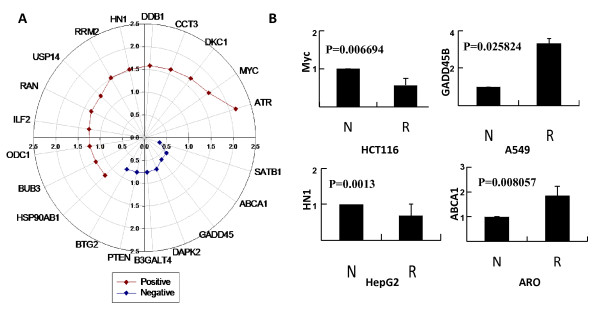
**Results of confirmatory qRT-PCR on selected genes**. (A) Polar plot of qRT-PCR validation results of selected genes highly correlated with REGγ. Following qRT-PCR analysis of specific genes in REGγ knockdown or control cells, data averaged from three independent experiments were converted into relative fold changes. Fold change values greater than one was shown as positive correlation (brown) and values less than one represented negative correlation (blue). Genes were arranged in different theta (θ) and radius represent the qRT-PCR fold change values from 0 to 3. Only data consistent with prediction were shown (see Table 2). (B) Representative qPCR validation experiments. Quantitative RT-PCR was performed in paired cancer cell lines (shN and shR) with differential levels of REGγ expression. Subsets of qRT-PCR results showed the actual expression differences of a specific gene in these cell lines (results were average from three independent experiments). The relative PCR values (Table 2) in shN (usually normalized as 1) were divided by the values in shR cells to yield fold changes as shown in Figure 5A. A value greater than 1 indicates a positive correlation with REGγ.

The relative expression ratios of each specific gene in shN and shR cancer cell lines were calculated from average of three independent experiments and the data with statistically significant changes (*p <*0.05) between shN and shR cells were plotted (Figure 5A and 5B). The expression of most genes (66.7%, n = 20) in at least one cell line with or without REGγ knockdown was consistent with predicted correlation to REGγ levels (Table 2).

**Figure 6:**
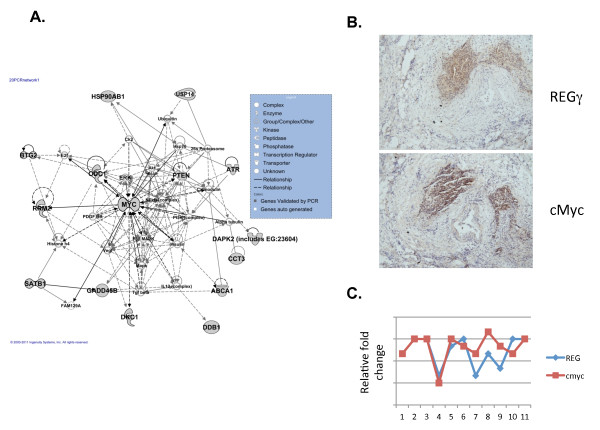
**Ingenuity network analysis of genes validated by qRT-PCR**. (A) Each node represents one gene and different shapes indicate a distinct function shown with the inlet. A solid line between genes indicates an interactive relationship, while a dotted line refers to potential relationship. A self-centered circle means a self interaction. (B) IHC analysis of Myc and REGγ in 11 colorectal cancer samples. All IHC were carried out using adjacent sections of cancer samples for either anti-REGγ or anti-Myc. The stained intensities were scored with double-blinded approaches following description in Figure 1. Relative fold levels were plotted with REGγ in blue lines/dots and Myc in brown lines/dots. (C) A representative IHC analysis of Myc and REGγ expression from sample #5 in (B).

All genes validated by RT-PCR were applied into Ingenuity system for core analysis. Network information showing the link among these REGγ correlated genes was displayed in Figure 6A. This analysis placed Myc as the hub of the interaction network and prompted us to perform further analysis on its biological significance. Interestingly, analysis of 11 colon cancer samples suggested significant positive correlation between cMyc and REGγ (Figure 6B and 6C). The p53 target, PTEN, was also observed in this network analyses, reinforcing the close correlation among REGγ, p53 pathway, and other cancer related pathways.

## Discussion

REGγ-proteasome system represents an emerging pathway recently recognized to be involved in cancer development. This study provides further links between REGγ and multiple cancer related pathways by a combination of bioinfomatic analysis and molecular biological approach.

To our knowledge, this is the first computational study so far in REGγ association with multiple cancers. We are also the first to demonstrate high expression of REGγ in lung and liver cancers despite that overexpression of REGγ in thyroid and colon cancer were reported [6,27]. Tissue array analyses of four different human cancers, including lung, colon, thyroid, and liver cancers, revealed significant increase of REGγ protein in over 50% of these cancer samples. Bioinformatic analysis of human microarray gene expression profiles indicates that REGγ gene expression is also increased in most of these human cancers, providing new evidence that REGγ-proteasome pathway may be involved in the development of multiple cancers.

Computational analysis of datasets from thyroid cancer with thyroid non-cancer disease and liver cancer with HCC clinical stage information indicated a potential correlation of gradual increase of REGγ level with cancer stages (Figure 3A). Although the sample size and numbers are relatively small, the results suggest a potential of REGγ as a prognostic cancer marker and hinted some molecular mechanisms linking REGγ to development of cancers toward later stages or malignancy.

Our meta-analysis disclosed significant correlation between REGγ and many genes in cancer and cancer related pathways from ingenuity analysis, including colorectal cancer, lung carcinoma, sarcoma, lymphoma, tumorigenesis, cell division and apoptosis related pathways etc. Importantly, genes downstream of the previously identified REGγ regulated proteins [24,25], p53, was found highly correlated with REGγ expression. Despite that p53 mutation in different cancer may complicate the correlation status of its downstream target genes with REGγ, the overall high correlation values strongly support the previous finding that REGγ-mediated regulation of p53 may play an important role in cancer development. Annotation analysis indicated significant correlation of REGγ with many different proteasome components, suggesting that REGγ may be elevated and function together with other proteasome complexes. Recent studies have indicated important roles of the ubiquitin-mediated protein degradation pathway in cancer and application of proteasome inhibitors as a promising anti-cancer therapeutic approach [28]. An additional interesting finding is that numerous REGγ-correlated genes are involved in metabolism, particularly in energy metabolism (Additional file 8: Table S7). The link between cell metabolism and cancers has been well documented [29]. The result concurs a recent notion that cancer cell metabolisms are controlled by oncogenes and tumor suppressor genes [30].

The mathematical approaches of bioinformatics used in this study are quite standard [18]. To minimize the chance of acquiring false-positive results and ensure that most of the strong candidate genes are selected, we set rigorous criteria for all studies performed. The significance of our computational analysis has been underlined by laboratory validation experiments in 4 pairs of cancer cell lines in which differential expression of REGγ was created in the same background to facilitate correlation studies. Results from quantitative analysis of selected genes were largely consistent with predicted correlations, suggesting powerful combination of bioinformatics and molecular biological studies in disclosing potentially novel functions of REGγ-proteasome in cancer progression. It is not unlikely that REGγ could serve as a cancer marker, particularly for cancers with aggressive behavior.

Given that REGγ mainly functions as a proteasome activator to induce protein degradation, the biological links between REGγ and its correlated genes may reflect a result of direct or indirect regulation on transcription. Ingenuity analysis of validated gene network led our attention to the correlation between REGγ and Myc gene. Previous research documented overexpression of both genes in colorectal cancers [31-33]. Coincident with the largest amount of datasets and highest REGγ differential expression from colon cancers, the positive correlation between REGγ and Myc was validated specifically in HTC116-shN and -shR cells. Since Myc functions as a transcription factor, we searched REGγ promoter and found numerous Myc binding sites within 1.5 KB upstream REGγ transcriptional initiation site (data not shown). Yet we could not exclude the possibility that REGγ may target a negative regulator of Myc for degradation. Further experiments will be carried out to understand the molecular detail of these hypotheses. It is likely that elevated expression of Myc in certain cancer cells is one of the potential mechanisms contributing to higher expression of REGγ.

## Conclusions

This study provides REGγ expression profiles based on computational analysis of published microarray datasets and laboratory experiments on cancer samples. Data analysis links REGγ to multiple cancer-related pathways. Our results indicate potentially important roles of REGγ in multiple cancer types and implicate REGγ as a putative cancer marker.

## Abbreviations

GEO: Gene expression omnibus; IHC: Immunohistochemistry; HCC: Primary hepatocelluar carcinoma; PTC: Papillary thyroid carcinomas; ATC: Anaplastic thyroid carcinoma; IBD: Inflammatory bowel disease; CD: Crohn's disease; UC: Ulcerative colitis; PCC: Pearson's correlation coefficient.

## Competing interests

The authors declare that they have no competing interests.

## Authors' contributions

XL, ST, Pei-Z, JC, LC, and RC designed the study. JH, RC and ST performed bioinformatics and statistical data analyses. XL, RC, and LC drafted the manuscript. YZ, GW and Ping-Z carried out quantitative PCR experiments and data interpretation. YZ, LJ and YY performed IHC and Western blot analysis. All authors read and approved the final manuscript.

## Funding

This work was supported by National Institutes of Health (1R01CA131914) and Norman Hackerman Advanced Research Program (1082318401; PN004949-0012-2009). This manuscript was also funded in part by the National Natural Science Foundation of China (30811120435, 30870503, 81071657); the Science and Technology Commission of Shanghai Municipality (06DZ22923, 11DZ2260300, 10JC1404200, 09ZZ41); the National Basic Research Program (2009CB918402, 2011CB504200); and the East China Normal University short-term oversee training program.

## Pre-publication history

The pre-publication history for this paper can be accessed here:

http://www.biomedcentral.com/1471-2407/12/75/prepub

## Supplementary Material

Additional file 1 **Table S2. A summary of 49 datasets collected for preliminary filtering**. Cancer types were identified according to the sample origin disclosed. Accession numbers were acquired from GEO. "Sample size" included all samples from cancer, disease and controls. Click here for file

Additional file 2 **Table S3. Datasets selected for analyzing genes differentially expressed A) **All cancer datasets used for two sample tests. **B) **All non-cancer disease datasets used for two sample tests. **C) **Four datasets with staged information for two sample tests. (XLS 35 kb). Click here for file

Additional file 3 **Table S1. Detailed IHC information of REGγ expression in multiple human cancers**. REGγ expression status was scored according to description in Materials & Methods. Overexpression rate of REGγ in each cancer was calculated based on the number of cases scored ++ and above. Pathological grading and histological information are provided for lung cancer (A), colon cancer (B), thyroid cancer (C) and liver cancer (D). Click here for file

Additional file 4 **Table S4. Information of 13 datasets for correlation analysis**. The "Critical Value for test of PCC in two tails" is variable depending on sample size of each dataset. The "Top % selected" was arbitrarily set. Click here for file

Additional file 5 **Table S5. Genes in p53 pathway following primary correlation analysis**. The twenty nine p53 regulated genes were screened following a pilot, less stringent correlation criteria (PCC +/- 0.6; binomial coefficient as 1). Predicted correlation in datasets was shown. Click here for file

Additional file 6 **Table S6. Ingenuity pathway analysis of genes highly correlated with REGγ**. The "Function Annotation" column shows the annotated pathways from Ingenuity knowledge base. "Molecules" refer to genes in each pathway. Pathways were ordered according to p-values. Click here for file

Additional file 7 **Figure S1. Features of genes highly correlated to REGγ following PCC and ingenuity analysis**. **(A) **REGγ highly-correlated genes shared in different cancers. The number in X axis at the bottom of each column represents the number of cancer types sharing REGγ highly-correlated genes. Y axis refers to the percentage of REGγ highly-correlated gene shared in cancers. **(B) **Top 15 significant pathways in Ingenuity analysis of genes highly correlated with REGγ. The X- axis shows the bio-function annotation for each of the 15 pathways. The black bars (# molecules) corresponding to Y-axis on the left represent the number of genes in each pathway. The crossed curve corresponding to Y-axis on the right shows the p-value in logarithm based on 10. The straight line refers to a cutoff for significant p-value. Click here for file

Additional file 8 **Table S7 KEGG pathway analysis of genes highly correlated with REGγ**. "KO" indicates the pathway map ID in the KEGG pathway database. "Gene Amount" represents the Gene number in each pathway form input genes. Click here for file

Additional file 9 **Figure S2 Protein-Protein interaction network of genes highly correlated with REGγ**. Nodes of sphere stand for proteins. Colored lines represent different types of protein-protein relationships including: green for neighborhood, red for gene fusion, blue for co-occurrence, black for co-expression, purple for experiments validated, light blue for databases, yellow for text-mining, and gray lines for homology. Length of the lines stands for the score of functional-link. Click here for file

Additional file 10 **Figure S3 RNA interference against REGγ significantly attenuated REGγ expression and function in different cancer cell lines**. Different cancer cell lines were generated by stably integrating a control shRNA or an shRNA specifically targeting REGγ (in A549, ARO and HCT116). RNA interference were also performed by transiently transfecting control (siN) and synthetic siRNA targeting REGγ (siR) to HepG2 cells. Resulted cells normally expressing REGγ (shN/siN) or with REGγ depletion (shR/siR) showed expressional and functional differences as demonstrated by change of p21, the known REGγ target. Click here for file

Additional file 11 **Table S8 Primers used in RT-PCR validation analysis**. Sequences of gene-specific primer sets for RT-PCR analysis were displayed. Click here for file
