# Supplementary material for: REGγ is associated with multiple oncogenic pathways in human cancers
Source: BMC Cancer. 2012 Feb 23;12:75. doi: 10.1186/1471-2407-12-75 (PMC3350384; doi:10.1186/1471-2407-12-75)
Supplement: Additional file 5 — Table S5. Genes in p53 pathway following primary correlation analysis. The twenty nine p53 regulated genes were screened following a pilot, less stringent correlation criteria (PCC +/- 0.6; binomial coefficient as 1). Predicted correlation in datasets was shown. [file 1471-2407-12-75-S5.DOC]

| Gene Symbol | Description | REGγ Correlation | | Reference |
| --- | --- | --- | --- | --- |
|  |  | (Prediction) | |  |
|  |  | Positive | Negative |  |
| BTG2* | BTG family, member 2 | 1 | 4 | [6] |
| HSP90AB1* | heat shock protein 90kDa alpha (cytosolic), class B member 1 | 5 | 0 | [20] |
| PTEN* | phosphatase and tensin homolog | 1 | 5 | [2] |
| RRM2* | ribonucleotide reductase M2 | 7 | 0 | [24] |
| TP53I3* | tumor protein p53 inducible protein 3 | 3 | 1 | [3] |
| BIRC5 | baculoviral IAP repeat-containing 5 | 9 | 1 | [17] |
| CCNG1 | cyclin G1 | 2 | 0 | [8] |
| CDC25C | cell division cycle 25 homolog C (S. pombe) | 3 | 1 | [18] |
| DNMT1 | DNA (cytosine-5-)-methyltransferase 1 | 6 | 0 | [19] |
| EFNA1 | ephrin-A1 | 3 | 3 | [28] |
| FANCC | Fanconi anemia, complementation group C | 1 | 4 | [15] |
| FEV | FEV (ETS oncogene family) | 1 | 5 | [27] |
| FXYD3 | FXYD domain containing ion transport regulator 3 | 3 | 3 | [9] |
| HRAS | v-Ha-ras Harvey rat sarcoma viral oncogene homolog | 5 | 1 | [7] |
| HSPA8 | heat shock 70kDa protein 8 | 3 | 0 | [21] |
| LRDD | leucine-rich repeats and death domain containing | 0 | 1 | [11] |
| MLH1 | mutL homolog 1, colon cancer, nonpolyposis type 2 (E. coli) | 6 | 0 | [5] |
| MMP2 | matrix metallopeptidase 2 (gelatinase A, 72kDa gelatinase, 72kDa type IV collagenase) | 0 | 8 | [10] |
| NDRG1 | N-myc downstream regulated 1 | 3 | 3 | [4] |
| PCNA | proliferating cell nuclear antigen | 6 | 1 | [3] |
| PERP | PERP, TP53 apoptosis effector | 2 | 0 | [13] |
| PMS2 | PMS2 postmeiotic segregation increased 2 (S. cerevisiae) | 1 | 1 | [5] |
| PPM1D | protein phosphatase, Mg2+/Mn2+ dependent, 1D | 2 | 0 | [26] |
| SH2D1A | SH2 domain containing 1A | 0 | 5 | [14] |
| THBS1 | thrombospondin 1 | 0 | 6 | [25] |
| TNFRSF10B | tumor necrosis factor receptor superfamily, member 10b | 3 | 3 | [16] |
| VDR | vitamin D (1,25- dihydroxyvitamin D3) receptor | 0 | 7 | [12] |
| YWHAQ | tyrosine 3-monooxygenase/tryptophan 5-monooxygenase activation protein, theta polypeptide | 7 | 0 | [22, 23] |
| GYPC | glycophorin C (Gerbich blood group) | 1 | 8 | [4] |
