# Supplementary figures and images for: REGγ is associated with multiple oncogenic pathways in human cancers
Source: BMC Cancer. 2012 Feb 23;12:75. doi: 10.1186/1471-2407-12-75 (PMC3350384; doi:10.1186/1471-2407-12-75)

## Slide 1
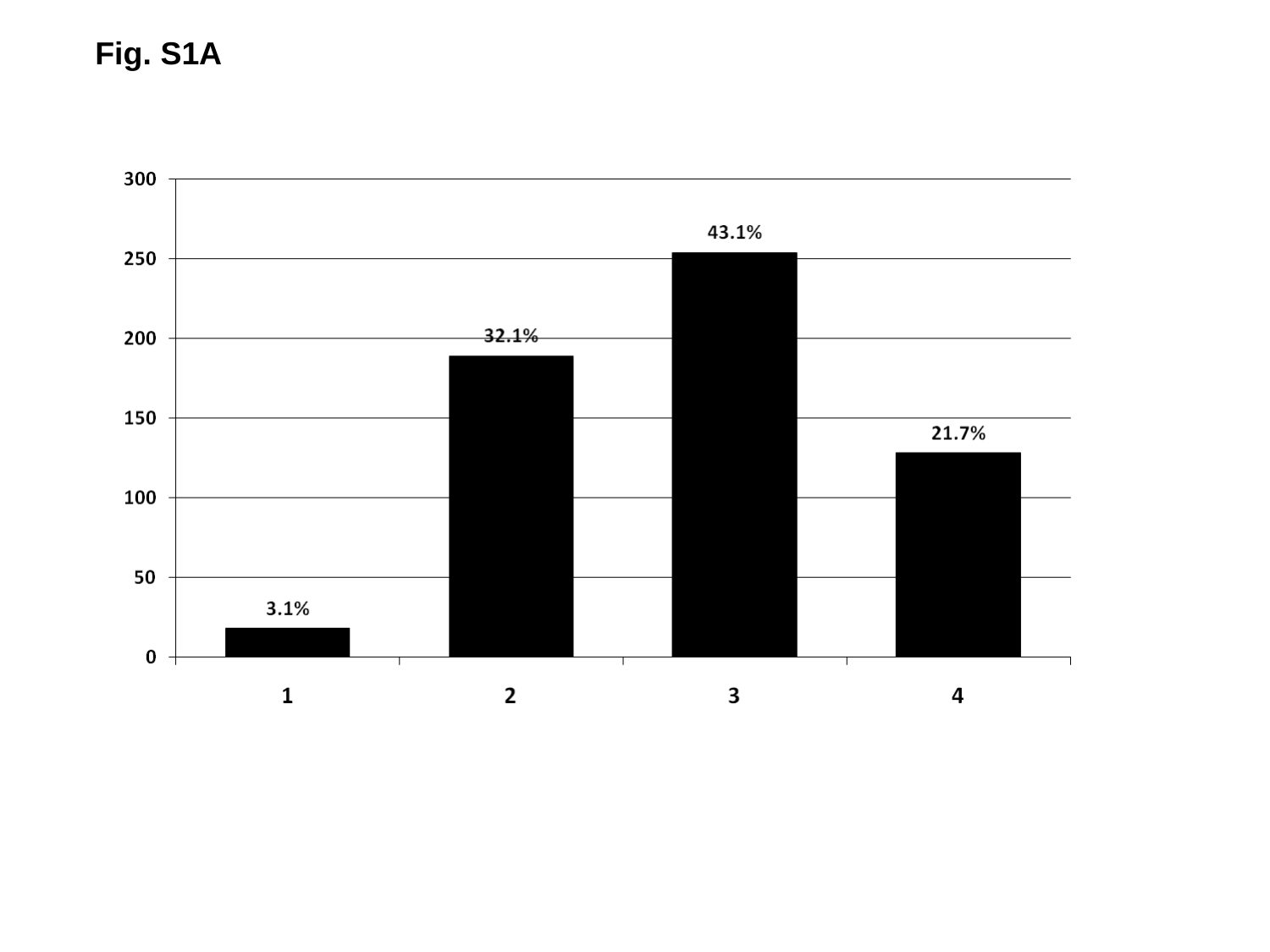

Fig. S1A

## Slide 2
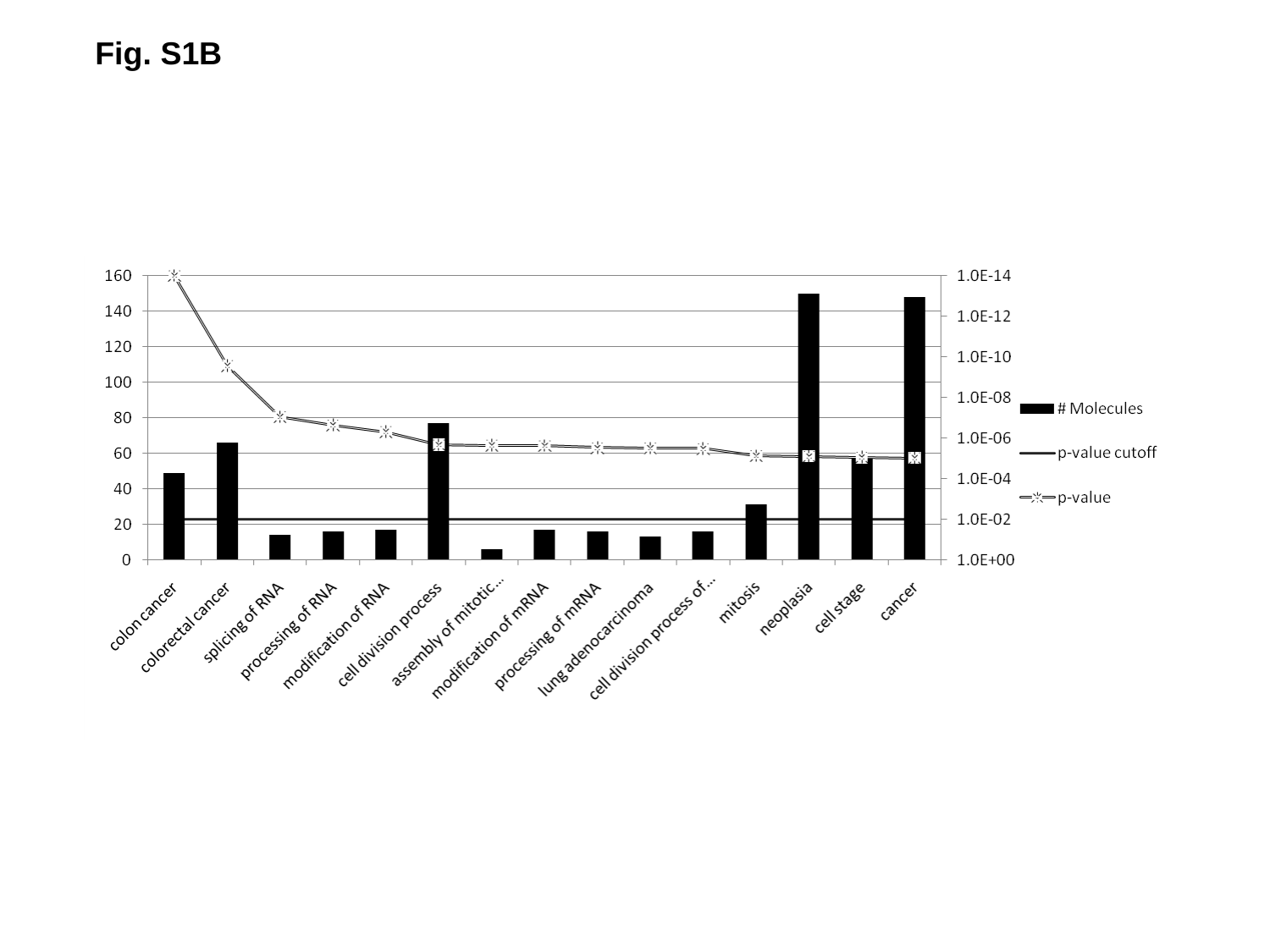

Fig. S1B

Supplement: Additional file 7 — Figure S1. Features of genes highly correlated to REGγ following PCC and ingenuity analysis. (A) REGγ highly-correlated genes shared in different cancers. The number in X axis at the bottom of each column represents the number of cancer types sharing REGγ highly-correlated genes. Y axis refers to the percentage of REGγ highly-correlated gene shared in cancers. (B) Top 15 significant pathways in Ingenuity analysis of genes highly correlated with REGγ. The X- axis shows the bio-function annotation for each of the 15 pathways. The black bars (# molecules) corresponding to Y-axis on the left represent the number of genes in each pathway. The crossed curve corresponding to Y-axis on the right shows the p-value in logarithm based on 10. The straight line refers to a cutoff for significant p-value. [file 1471-2407-12-75-S7.PPT]

## Slide 1
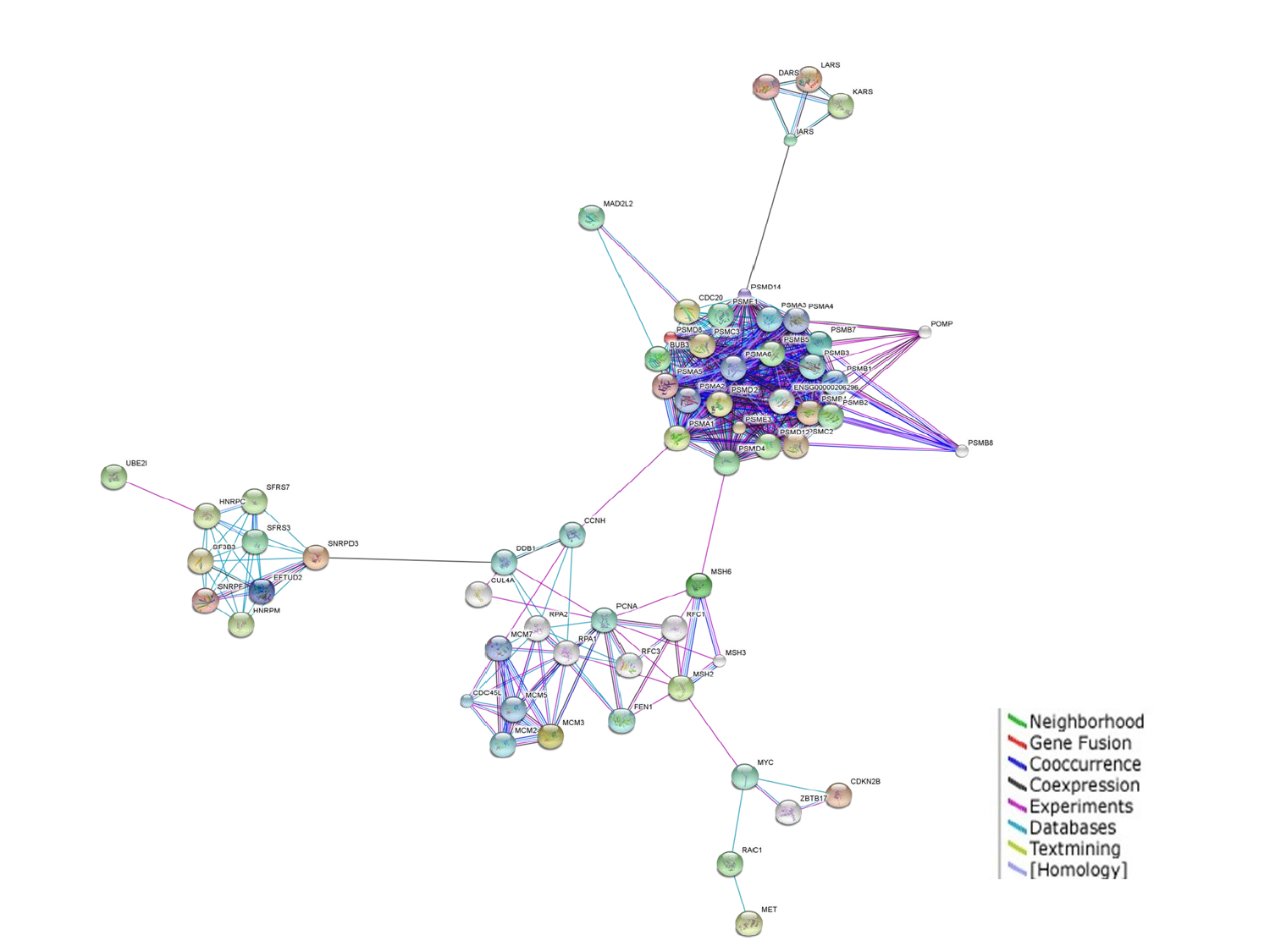

Supplement: Additional file 9 — Figure S2 Protein-Protein interaction network of genes highly correlated with REGγ. Nodes of sphere stand for proteins. Colored lines represent different types of protein-protein relationships including: green for neighborhood, red for gene fusion, blue for co-occurrence, black for co-expression, purple for experiments validated, light blue for databases, yellow for text-mining, and gray lines for homology. Length of the lines stands for the score of functional-link. [file 1471-2407-12-75-S9.PPT]

## Slide 1
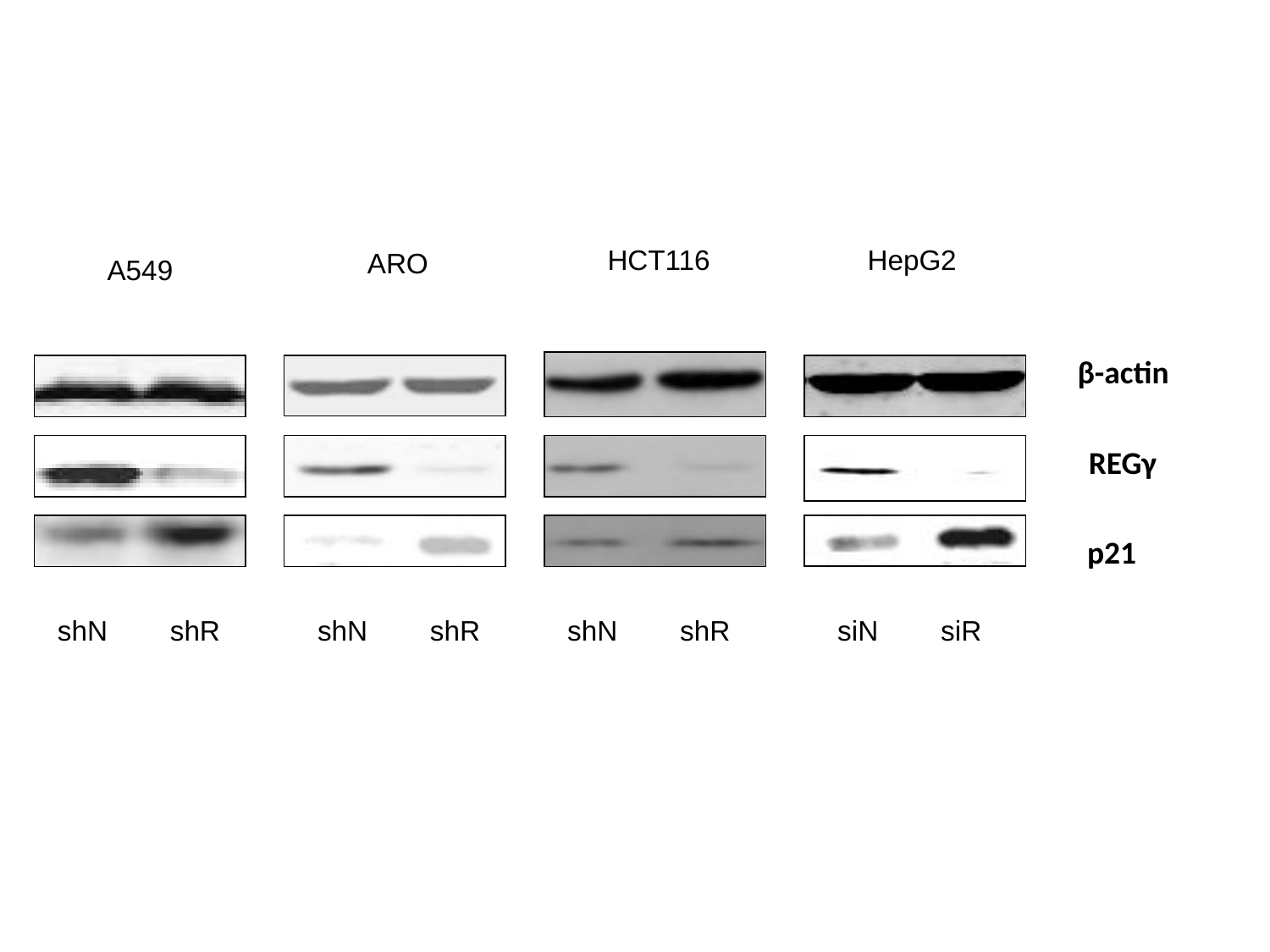

HCT116
HepG2
ARO
A549
β-actin
REGγ
p21
shN shR
shN shR
shN shR
siN siR

Supplement: Additional file 10 — Figure S3 RNA interference against REGγ significantly attenuated REGγ expression and function in different cancer cell lines. Different cancer cell lines were generated by stably integrating a control shRNA or an shRNA specifically targeting REGγ (in A549, ARO and HCT116). RNA interference were also performed by transiently transfecting control (siN) and synthetic siRNA targeting REGγ (siR) to HepG2 cells. Resulted cells normally expressing REGγ (shN/siN) or with REGγ depletion (shR/siR) showed expressional and functional differences as demonstrated by change of p21, the known REGγ target. [file 1471-2407-12-75-S10.PPT]
