# Supplementary material for: REGγ is associated with multiple oncogenic pathways in human cancers
Source: BMC Cancer. 2012 Feb 23;12:75. doi: 10.1186/1471-2407-12-75 (PMC3350384; doi:10.1186/1471-2407-12-75)
Supplement: Additional file 11 — Table S8 Primers used in RT-PCR validation analysis. Sequences of gene-specific primer sets for RT-PCR analysis were displayed. [file 1471-2407-12-75-S11.PDF]

Gene Symbol	F	R
DAPK2	CCCGCCGATTGTATGTTC	GCTCTGCCGCTTCTTGAT
ABCA1	CTGTATGGGTGGTCAATC	AACACGGACTTCAGGATA
GADD45B	GCGCAGAAGATGCAGACG	ACACCCGCACGATGTTG
SATB1	CAGGCCAAAGGGCTAATC	GCGACCATTGTTCGGGAG
RRM2	CTTGGTGGAGCGATTTAG	CCCAGTCTGCCTTCTTCT
BUB3	GTAATGATGGGACTACGC	ACATGGTGACTTGGGTTT
USP14	TGCCGCTCTACTCCGTTAC	CGTTCCTCCTTTCACCAT
ATR	GCTAACAGGTCCGAGTGG	TGATAATAATAGCTGTCCC
CDK1	ACCATACCCATTGACTAAC	ATCCTGCATAAGCACATCC
STARD8	GCCACAGAGCATTCAGCA	CAGGCGAGGTCTCATTCA
UNC13A	GGACAAGCGAACTGACAAA	TAGTAAACCTTCCAGGCATC
PTEN	AGAAAGACTTGAAGGCGTA	GCTGTGGTGGGTTATGGT
B3GALT4	AACGCCATTCGGGCTTCG	GGGTGAGGTTGCGGTAGGA
ILF2	GGGAACAAAGTCGTGGAA	ATGTCGGATGGCTGCTAA
ODC1	GTGGGTGATTGGATGCTC	TCCTCTACTTCGGGTGGG
HSP90AB1	GTCTTCTGCTGGAGGTTC	AATAAAGGGTGATGGGAT
MYC	CCGAAGGGAGAAGGGTGT	CTGCGACGAGGAGGAGAA
BTG2	CATCATCAGCAGGGTGGC	CCCAATGCGGTAGGACAC
